# Clustering of non-medical risk factors and the association with duration of social care in pregnant women in highly vulnerable circumstances

**DOI:** 10.1093/eurpub/ckaf062

**Published:** 2025-04-27

**Authors:** Kajal S C Mohabier, Lizbeth Burgos-Ochoa, Johanna P de Graaf, Eric A P Steegers, Loes C M Bertens

**Affiliations:** Department of Obstetrics and Gynaecology, Erasmus University Medical Centre Rotterdam, Rotterdam, The Netherlands; Department of Methodology and Statistics, Tilburg University, Tilburg, The Netherlands; Department of Obstetrics and Gynaecology, Erasmus University Medical Centre Rotterdam, Rotterdam, The Netherlands; Department of Obstetrics and Gynaecology, Erasmus University Medical Centre Rotterdam, Rotterdam, The Netherlands; Department of Obstetrics and Gynaecology, Erasmus University Medical Centre Rotterdam, Rotterdam, The Netherlands

## Abstract

Pregnancy can be considered a window of opportunity to help pregnant women optimize the circumstances they live in. Within the Mothers of Rotterdam study, pregnant women in highly vulnerable circumstances received standard social care or targeted social care to improve their circumstances. Women in this study had many combinations of non-medical risk factors contributing to their vulnerable circumstances. Here, the aim is to study the association between different combinations of non-medical risk factors and duration of care. Existing non-medical risk factors, assessed with a vulnerability checklist, were clustered using Latent Class Analysis (LCA). Linear regression was used to examine the relationship with duration of social care. The model was adjusted for maternal age, deprived neighbourhood, and type of social care. Four vulnerability classes were identified among 840 women and were labelled complex (9%), educational (24%), social network (12%), and financial vulnerability (55%). In the unadjusted model, all three classes showed a significant longer duration of social care compared to the financial vulnerability class. After adjustment, only the longer duration of care of the social network vulnerability class remained statistically significant. The four identified vulnerability classes illustrate that even within a group of women in highly vulnerable circumstances, subgroups of vulnerability exist. The vulnerability classes were identifiable through different combinations of non-medical risk factors and are all, associated with different durations of social care. These findings help to understand, and plan for, the requirements of social care for women in highly vulnerable circumstances.

## Introduction

Pregnancy can be considered a perfect window of opportunity for dealing with experienced problems and optimizing the circumstances someone lives in. The premise of new life can be a strong motivator to adopt healthier behaviours and optimize living circumstances [1, 2]. In addition to increased motivation for change and receiving care during pregnancy, the chances for a healthy growth and development of the (unborn) child is optimized [3–6]. There is a vast body of literature illustrating the importance of intervening with unfavourable circumstances in the first 1000 days of life, including pregnancy, to optimize health, well-being and opportunities for both mother and child [5, 7–11].

The Mothers of Rotterdam study evaluates the impact of two types of social care for pregnant women in highly vulnerable circumstances in Rotterdam [12, 13]. Both types of social care are able to support their clients with problems from most life domains and refer to specialized care when needed. They differ in their approach; where Standard Social Care (SSC) is part of regular social care and focusses on those problems indicated by the client, Targeted Social Care (TSC) is specifically designed for pregnant women, and holds a more proactive approach to providing care. By design, TSC spends more time with their clients and lasts until the second birthday of the child. The most pronounced difference between SSC and TSC can be measured with the duration of social care provided. However, just comparing the differences in duration seems inadequate since the underlying problems faced by a client largely influences the time spent to deal with it [7, 14, 15]. This becomes even more complex when a client faces multiple problems. Learning how different combinations of non-medical risk factors are associated with duration of care, can help in the understanding of the impact of dealing with these problems in daily life. Moreover, the identification of subgroups with vulnerable populations can help design and tailor effective social care strategies to better fit the care needs of this population.

Taking into account the different combinations of non-medical risk factors could help the comparison of duration of care between different care approaches. In the MoR study, women report a median of six problems over four life domains, in many different combinations [12]. This complexity needs more sophisticated analytical methods like latent class analysis (LCA) to help identify subgroups in the data. The aim was to study the association between the vulnerability classes, identified with LCA, and duration of social care. Because the duration of social care is likely to differ between social care approaches, type of social care was considered an important covariate in the analyses.

## Methods

### Study population

Data for this study comes from the MoR study, in which social care for pregnant women in highly vulnerable circumstances is evaluated [12, 13]. Recruitment for the MoR study (January 2016–December 2020) was open to all pregnant women who resided in Rotterdam, who were considered to be highly vulnerable, and who were referred to, or applied for, social care within the MoR program. Referring parties included obstetric professionals, social workers and the social network of the pregnant woman. More details about the recruitment can be found in the protocol and baseline description of the MoR study [12, 13]. To identify existing non-medical risk factors, a ‘vulnerability checklist’ was used. This checklist was developed by the social care organization and used in daily care practice. Social care professionals evaluated eligibility and drafted a care plan in the home environment of the pregnant woman. Based on the social care plan, the checklist was filled out by the social care professional. The checklist consists of 47 non-medical risk factors divided over eight life-domains, and are known to influence self-sufficiency, well-being, and health of the pregnant woman and/or the health of her (unborn) child (see Supplementary Appendix S1). Pregnant women facing a minimum of three non-medical risk factors over at least two life-domains on this checklist were considered eligible for receiving care and participation in the study. Social care provision to these women was independent of their participation in the MoR study. In the first three months it was not uncommon for social care to be transferred to other specialized organizations when needed. The Mothers of Rotterdam study was carried out in accordance with the relevant guidelines and regulations. The Erasmus Medical Centre Ethics Committee (ref. no. MEC-2016-012) approved the study and informed consent was obtained from all individual participants included in the study.

### Data collection, main outcome measure, and determinants

For all participating women, baseline maternal and social characteristics were retrieved from the application forms, including the vulnerability checklist, as an integral part of the study. The main outcome measure was duration of social care, defined as the time difference between the start and the end of social care, measured in months. Sixty-nine participating women still received social care at the moment of data extraction. In these cases, the date of data extraction was used as a fictitious end date (i.e. 1 August 2021).

The main determinants are the items from the vulnerability checklist, grouped into classes of vulnerability using LCA (also see statistical analyses) [16]. Items on the vulnerability checklist were used for this analysis. Due to their high similarity, five items were merged into two new ones; the items homeless and couch-crashing were combined into ‘homelessness’, and three items on education were combined into ‘only primary education or less’. The item ‘lack of baby layette’ was only considered relevant when present after 34 weeks of pregnancy. This resulted in 44 items from the vulnerability checklist used in the LCA.

The other baseline maternal and social characteristics included maternal age (<20, 20–29, 30–39, and ≥40 years), type of social care (TSC or SSC), and living in a deprived neighbourhood (yes, no); defined according to the guidelines of the Dutch Healthcare Authority [17] and assigned via the residential postal of the woman.

### Statistical analyses

Baseline maternal and social characteristics were tabulated for all participating women receiving social care, for the total sample and stratified by vulnerability class.

#### Latent class analysis

LCA was used to identify vulnerability classes based on the items of the vulnerability checklist. LCA is a statistical technique to identify mutually exclusive, and exhaustive subgroups of people based on observed variables [16, 18, 19]. These classes are considered latent since they are not directly measurable but classified based on the patterns in responses of the indicator items. A probabilistic approach is used to find the best-fitting model and assign each individual a probability of belonging to class membership. The model with the lowest values for the Bayesian Information Criterion (BIC) and the consistent Akaike Information Criterion (AIC) is then selected as the best-fitting model. To improve fit, maternal age, living in a deprived neighbourhood and type of social care were added to the LCA as covariates. The main study outcome was added to the model as a distal outcome. This analysis step, known as classify-analysis, was proposed by Bray *et al.* [20, 21] and aims to circumvent the production of attenuated estimates by potential measurement errors. Each woman was assigned to one of the four classes based on maximum probability. A more elaborate description of the LCA process and considerations can be found in Supplementary Appendix S2.

##### Regression analyses

Linear regression models were fitted to examine the relation between the identified vulnerability classes and duration of social care. The duration of social care was log-transformed to better approximate a linear distribution of the outcome. The most prevalent vulnerability class served as reference category. The regression model was then adjusted for maternal age, living in a deprived neighbourhood, and type of social care. Interaction terms were included to examine the potential moderating effects of vulnerability class*type of social and vulnerability class*neighbourhood.

Two sensitivity analyses were performed. One excluding cases which ended care in the first three months, since a transfer of care to more specialized organizations is more common in this period. This model showed very similar results to the main analysis but with smaller confidence bounds (Supplementary Appendix S3). Therefore, the results of these analyses were presented as the main results. The second sensitivity analyses excluded the women who still received care (Supplementary Appendix S3).

The threshold of statistical significance was set at *P* < 0.05. The LCA was conducted in R version 3.5.3., package poLCA., and the descriptive and linear regression analyses in SPSS version 28 [22].

## Results

Social care professionals assessed eligibility of 919 pregnant women applying for social care between January 2016 and December 2020, of which 862 pregnant women were included in the MoR study. For 22 of these women the vulnerability checklist was not complete and therefore excluded from the analyses, resulting in records for 840 participating women in the LCA analyses.

Baseline maternal and social characteristics of the MoR population, in total and stratified by vulnerability class are described in Table 1. Women were on average 28 years of age and 8% of them were aged under 20 years, 58% resided in a deprived neighbourhood, and 64% received TSC. The mean duration of social care was 11 months.

**Table 1.** Baseline maternal and social characteristics^a^

Class	Total sample	Complex	Educational	Social network	Financial
Number of women (%)	840 (100.0)	72 (8.6)	201 (23.9)	103 (12.3)	464 (55.2)

**Maternal age**					
Mean in years (SD)	27.5 (5.9)	29.1 (5.7)	27.8 (6.3)	29.5 (6.3)	26.7 (5.6)
<20	63 (7.5)	3 (4.2)	13 (6.5)	6 (5.8)	41 (8.8)
20–29	489 (58.2)	38 (52.8)	114 (56.7)	52 (50.5)	285 (61.4)
30–39	262 (31.2)	28 (38.9)	63 (31.3)	39 (37.9)	132 (28.4)
≥40	26 (3.1)	3 (4.2)	11 (5.5)	6 (5.8)	6 (1.3)
**Deprived neighbourhood**					
Yes	486 (57.9)	42 (58.3)	101 (50.2)	53 (51.5)	290 (62.5)
No	343 (40.8)	26 (36.1)	100 (49.8)	49 (47.6)	168 (36.2)
Missing	11 (1.3)	4 (5.6)	0 (0.0)	1 (1.0)	6 (1.3)
**Type of social care**					
Targeted Social Care (TSC)	535 (63.7)	55 (76.4)	130 (64.7)	65 (63.1)	285 (61.4)
Standard social care (SSC)	305 (36.3)	17 (23.6)	71 (35.3)	38 (36.9)	179 (38.6)
**Duration of social care**					
Mean in months (SD)	11.3 (9.1)	11.6 (8.9)	13.6 (10.9)	12.7 (9.0)	9.9 (8.0)
**Problematic life-domains**					
Pregnancy	482 (57.4)	42 (58.3)	85 (42.3)	77 (74.8)	278 (59.9)
Residence	385 (45.8)	**72 (100.0)**	43 (21.4)	54 (52.4)	216 (46.6)
Finance	640 (76.2)	**62 (86.1)**	107 (53.2)	**93 (90.3)**	**378 (81.5)**
Occupation	443 (52.7)	46 (63.9)	**143 (71.1)**	60 (58.3)	194 (41.8)
Parenting	122 (14.5)	15 (20.8)	7 (3.5)	74 (71.8)	26 (5.6)
Health	653 (77.7)	**72 (100.0)**	102 (50.7)	**101 (98.1)**	**378 (81.5)**
Social functioning	681 (81.1)	**67 (93.1)**	**199 (99.0)**	**87 (84.5)**	328 (70.7)
Safety	182 (21.7)	36 (50.0)	7 (3.5)	54 (52.4)	85 (18.3)

a Data displayed as frequency (%), unless otherwise stated. SD = standard deviation. For each vulnerability class, the numbers are presented in **bold** for the domains that are characteristic for that class.

**Figure 1.**
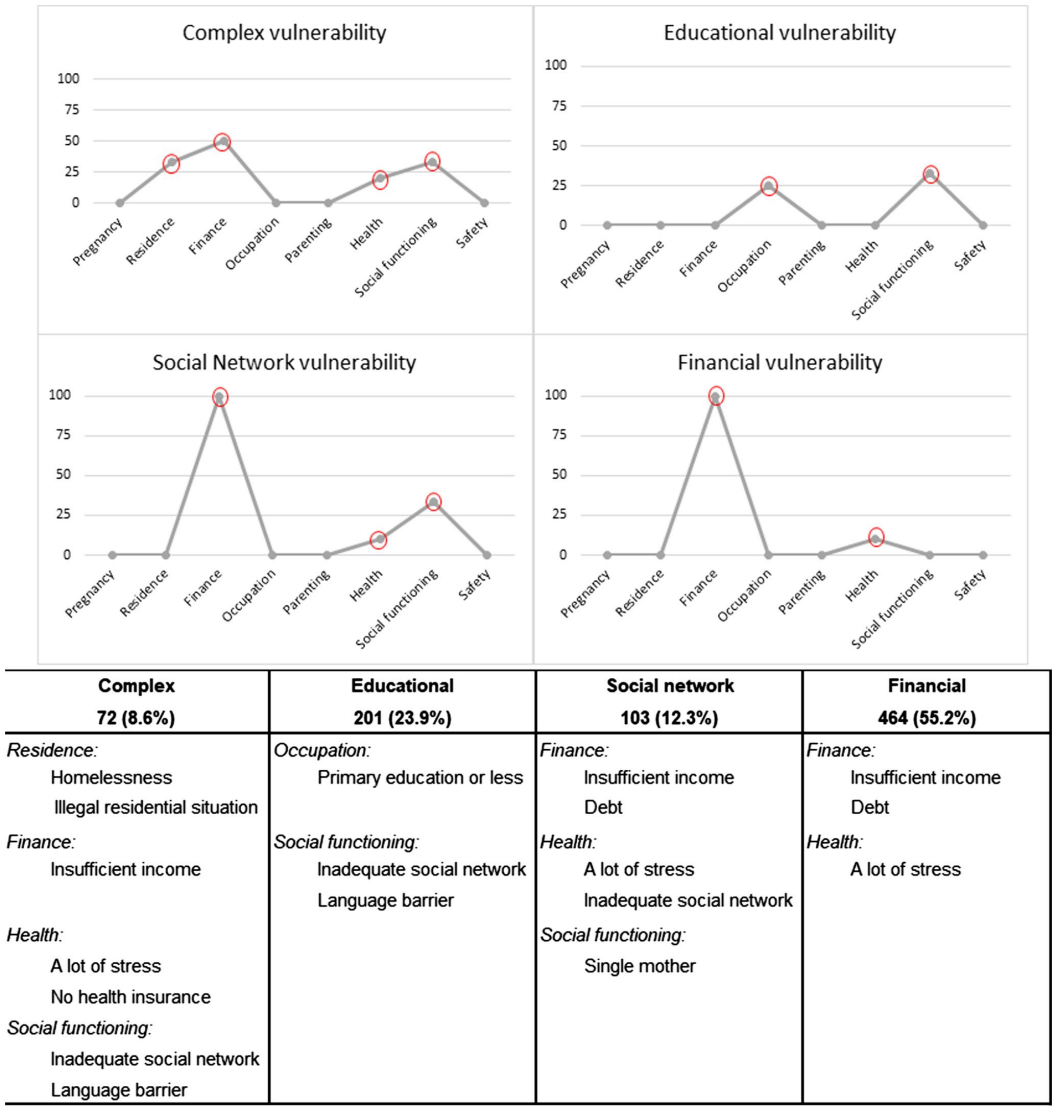
Characterizing life-domains and adjective problems per identified latent vulnerability class. This figure provides an overview of the characterizing life-domains and items per identified vulnerability class, which are labelled: complex, educational, social network, and financial. At the top of the figure, four point to point graphs visualize the chances of the presence of the items mentioned below, per life domain. For example, there is a 33% chance of a problem with homelessness or illegal housing situation in the complex vulnerability class. These chances are extracted from the LCA model and are therefore different from the observed percentages in Table 1. At the bottom of the figure, adjective problems per vulnerability class are tabulated.

For the LCA, a four-class model was considered the best-fitting model (BIC= 19686.56 and AIC= 18860.37, see Supplementary Appendix S2). The identified vulnerability classes were: complex vulnerability (9%), educational vulnerability (24%), social network vulnerability (12%), and financial vulnerability (55%). For each vulnerability class, several items from the vulnerability checklist are characteristic for that class (Fig. 1). Some items, like insufficient income, language barrier, and inadequate social network, are mentioned for multiple classes. The combinations of items, along with a dominance of certain life domains, is what makes the identified classes distinct.

Women in the complex and social network vulnerability classes were on average older than women in the educational and financial vulnerability classes (29 and 30 years compared to 28 and 27 years, respectively). TSC was provided to the majority of women; with 75% in the complex vulnerability class and 65% in the other classes. Compared to the other classes, women in the financial vulnerability class more often resided in a deprived neighbourhood and had the shortest duration of social care (10 months).

### Linear regression analyses

**Figure 2.**
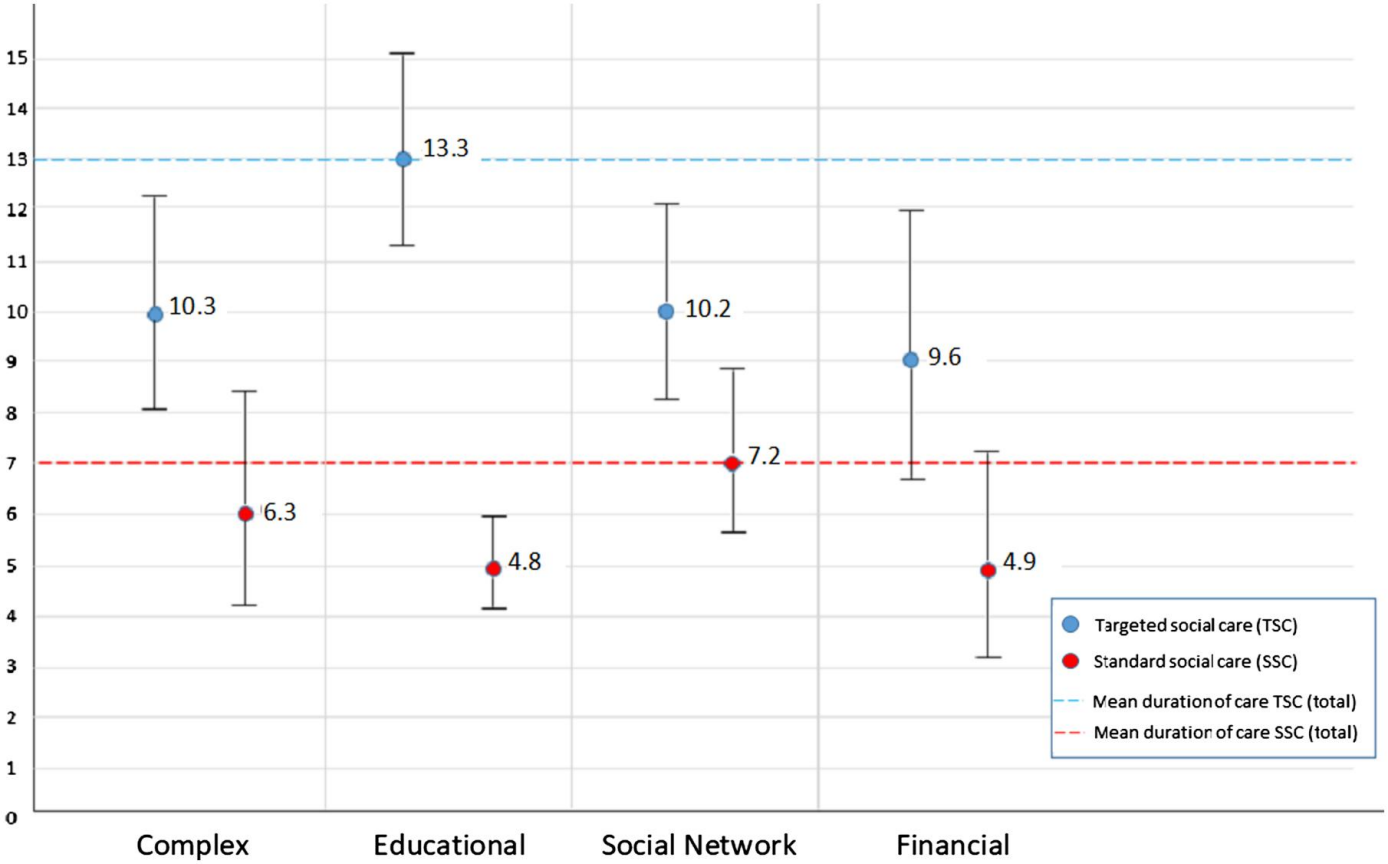
Mean duration of social care in months per class—stratified by type of social care. This figure provides an overview of the mean duration of social care in months per identified latent vulnerability class and stratified by type of social care (i.e. TSC and SSC). The figure shows the mean durations of TSC as blue dots with the 95% confidence intervals plotted around it. The blue broken line depicts the mean duration of social care for the total group of TSC. The figure shows the mean durations of SSC as red dots with the 95% confidence intervals plotted around it. The red broken line depicts the mean duration of social care for the total group of SSC.

As described in the method section, the initial sensitivity analyses are presented as main findings. A total of 144 women (17.1%), who ended their social care within the first three months, were excluded, leaving us with 696 women for the analyses. The vulnerability classes were similarly distributed in this sample: with 9% complex, educational in 24%, social network in 13%, and financial in 55%. Interaction terms with deprived neighbourhood with the classes were non-significant and removed from the final model. Table 2 shows the results of the linear regression, along with two examples to guide the interpretation of the model. The duration of social care in the complex, educational and social network vulnerability classes compared to the financial vulnerability class is visualized in Fig. 2.

**Table 2.** Linear regression analyses in 696 participants completing at least three months of care^a^

	Unadjusted regression		Adjusted regression	
	Effect estimate (β), 95%CI	*P*-value	Effect estimate (β), 95%CI	*P*-value
Intercept	0.981		0.522	
Class 1: Complex	0.082 (0.003; 0.161)	*0.041*	0.145 (−0.058; 0.347)	0.161
Class 2: Educational	0.122 (0.070; 0.175)	*<0.001*	−0.023 (−0.135; 0.089)	0.686
Class 3: Social network	0.096 (0.030; 0.162)	*0.005*	0.226 (0.082; 0.370)	*0.002*
Class 4: Financial	*Reference*		*Reference*	
Maternal age	*NA*		0.008 (0.003; 0.013)	*0.001*
Deprived neighbourhood^b^	*NA*		−0.093 (−0.150;−0.035)	*0.002*
Type of social care^c^	*NA*		0.260 (0.183; 0.337)	*<0.001*
Interaction type of social care—Class 1	*NA*		−0.147 (−0.384; 0.090)	0.223
Interaction type of social care—Class 2	*NA*		0.174 (0.033; 0.315)	*0.015*
Interaction type of social care—Class 3	*NA*		−0.215 (−0.395;−0.034)	*0.020*

a The outcome values, duration of care, are log transformed.

b Deprived = 1.

c Mothers of Rotterdam = 1; Interaction term neighbourhood-class showed *P* > 0.05 during backward selection procedures and was therefore excluded from the final model.

**Example 1:**

– Woman A with adversities identified in class 3, 21 years old, lives in a deprived neighbourhood, and receives TSC.

Duration of social care = 10 ^ (0.522 + 0.226*1 + 0.008*21 + −0.093*1 + 0.260*1 + −0.215*1*1) = 7.4 months.

– Woman B with adversities identified in class 3, 21 years old, lives in a deprived neighbourhood, and receives SSC.

Duration of social care = 10 ^ (0.522 + 0.226*1 + 0.008*21 + −0.093*1 + 0.260*0 + −0.215*0*1) = 6.7 months.

**Example 2:**

– Woman C with adversities identified in class 3, 32 years old, lives in a deprived neighbourhood, and receives TSC.

Duration of social care = 10 ^ (0.522 + 0.226*1 + 0.008*32 + −0.093*1 + 0.260*1 + −0.215*1*1) = 9.0 months.

– Woman D with adversities identified in class 2, 32 years old, lives in a deprived neighbourhood, and receives TSC.

Duration of social care = 10 ^ (0.522 + −0.023*1 + 0.008*32 + −0.093*1 + 0.260*1 + 0.174*1*1) = 12.5 months.

In the unadjusted model, all three classes showed a significant longer duration of care compared to the financial vulnerability. The original and two sensitivity analyses were very similar in the unadjusted analyses (Supplementary Appendix S3). After adjustment, only the association with social network vulnerability remained statistically significant. Maternal age and receiving TSC were both significantly associated with a longer duration of care (β 0.008, 95% CI: 0.003, 0.013 and β 0.260, 95% CI: 0.183, 0.337, respectively), while living in a deprived neighbourhood was associated with a shorter duration of care (β −0.093, 95% CI: −0.150, −0.035). Moreover, the associations for educational vulnerability and social network vulnerability with duration of care were significantly moderated by type of social care.

## Discussion

In this study, we identified four vulnerability classes within a group of pregnant women in highly vulnerable circumstances, which were labelled as complex, educational, social network and financial vulnerability. Each vulnerability class had differentiating combinations of problems. We found that the financial vulnerability class was associated with a shorter duration of care compared to the other classes. Next to the classes, type of social care, maternal age and living in a deprived neighbourhood were also associated with duration of care.

Although no prior research has identified subgroups within highly vulnerable populations, a recent study of Dutch pregnant women found that risk factors across multiple life-domains led to more adverse health outcomes compared to those experiencing no adversities or just in one domain [23]. This finding suggests that there is a continuous scale in degree of vulnerability, with greater need for care for the higher degrees of vulnerability. While our study underlines that different groups of vulnerability have different durations of care, we can only underscore that the least vulnerable class had a shorter duration of care. However, within the other three vulnerability classes, the duration of care no longer seems to increase with increasing complexity.

The type of problems faced by the women seems to influence duration of care; some problems are easier to address than others. For instance, financial problems can be addressed relatively easily and quickly as financial support programs are available through the municipality for individuals and families facing financial hardship or unemployment [24–26]. On the other hand, difficulties with social networks require more time and effort. The complex vulnerability class, notably, was characterized by problems such as homelessness and illegal residential situations. It is likely that these problems often result in multiple relocations, which increases the risk of care discontinuation.

Next to the problems faced, the type of social care was also associated with duration of care in our study. Which was expected, as TSC planned for longer duration of social care compared to SSC [13]. Next to the differences in planned amount of time spent with clients, logistical features of social care organizations may also play a role.

Maternal age and living in a deprived neighbourhood were associated with duration of care in a different way than expected. Generally, individuals tend to become more self-sufficient as they age [27]. In our study increasing maternal age was significantly associated with a longer duration of care. A reason could be that older women might have more children and thereby appreciating the extra help for a longer period or are better able to advocate their needs. We expected to find a longer duration of care for women living in a deprived neighbourhood, while we found the opposite. A considerable part of our population dealt with homelessness or stayed at the place of friends or family. Potentially, these women where now assigned to live in a non-deprived neighbourhood, thereby masking the true association between deprivation and duration of care. However, we don’t now the reasons for care (dis)continuation which complicates the interpretation of these associations.

### Strengths and limitations

A major strength of the MoR study is the relatively high number of pregnant women in highly vulnerable circumstances that were included into the study. Due to the high accessibility of the MoR program we were even able to identify women with an illegal status, language barriers and women with an inadequate social network, groups that are notoriously hard to reach.

To our knowledge, this study is the first to define (sub)groups within a group of pregnant women in highly vulnerable circumstances using LCA. Despite the complexity of our data, we were able to organize the data into four classes. Also, by using this approach, more emphasis is put on the combination of problems and risk factors rather than on the individual risk factors themselves.

For the identification of the vulnerability classes, we relied on the items scored on the Vulnerability checklist. This checklist is not designed for research purposes and when an item is not filled, we did not know whether it was not present or just not discussed, and thus missing. Also, some items like smoking, alcohol and drug use could be underreported because of social desirability.

Unfortunately, we did not have data available on other factors, like presence of a partner in the household, that could have influenced the duration of care or the vulnerability classes.

Even though the duration of care can be used as a proxy for care needs, it is not the same, and care need is much more complex. Uptake and availability of care also play an important role. Also, more subjective processes as distrust or unsafe and unstable living circumstances largely influences the ability of a person to uptake and continue care. In our data, this was observed in women in the complex vulnerability class, who face the highest complexity in problems, while this did not translate into a longer duration of care. Reasons for discontinuation of care were not always available, however, understanding these reasons could contribute to a deeper understanding of the impact and challenges faced by these women.

### Future implications

Identifying non-medical risk factors are important for developing tailored interventions to support (sub)populations of highly vulnerable (pregnant) women [6, 28]. Care providers should be thorough in their process of identification, since we show that the specific combinations of non-medical risk factors are of influence on the duration of care, and likely also the care need.

By making use of the window of opportunity created by the pregnancy, women that otherwise tend to avoid care, might now be motivated to take up care [1, 14, 15, 28, 29]. Especially in these cases it is crucial that all problems are identified and included in the social care plan. By doing so, these women are helped with their current problems and can start building a safe and healthy basis for the future with their child. Eventually, this has the potential to mitigate the negative intergenerational effects of poverty and other deprivation related problems [5, 7, 8, 11]. Future research could update our found vulnerability classes by including protective factors and coping strategies into the model. Also, other relevant outcomes like health and well-being could be considered.

## Conclusion

In this study, we identified four vulnerability classes in a group of pregnant women in highly vulnerable circumstances, each characterized by different combinations of non-medical risk factors, and associated with different durations of care. By identifying these classes and their relationship to care duration, we gained a more nuanced understanding of the challenges faced by people in vulnerable circumstances. Recognizing these nuances, care interventions can be developed or tailored to better support women in highly vulnerable circumstances. To be able to do so, it is essential for caregivers to take time to observe, listen and communicate with individuals receiving social care, particularly those in highly vulnerable situations.

## Supplementary Material

ckaf062_Supplementary_Data

## Data Availability

The datasets used in the current study are available from the corresponding author on reasonable request.
