# Supplementary material for: Clustering of non-medical risk factors and the association with duration of social care in pregnant women in highly vulnerable circumstances
Source: Eur J Public Health. 2025 Apr 27;35(3):521–7. doi: 10.1093/eurpub/ckaf062 (PMC12199338; doi:10.1093/eurpub/ckaf062)
Supplement: ckaf062_Supplementary_Data [file ckaf062_supplementary_data.zip › ckaf062_Supplementary_Data/ejph-2024-06-om-0381-File007.docx]

Appendix 2. Description Latent Class Analysis process

Latent Class Analysis (LCA) is a statistical technique to identify mutually exclusive, and exhaustive subgroups of people (latent classes) on the basis of observed variables.^1^ These classes, as categories of a latent variable, cannot be directly measured but through the patterns in the responses of the indicator items. LCA uses a probabilistic approach to find the best fitting model and assign each individual a probability of belonging to each subgroup (class membership); LCA has been shown to be a useful technique for identifying qualitatively different population subgroups in a variety of disciplines, including epidemiology and public health.

To relate the identified latent classes to a distal outcome (i.e. duration of social care), the improved classify-analysis proposed by Bray et al. was used in this study to circumvent the production of attenuated estimates. The second aim of the study is to relate the identified latent classes to a distal outcome, duration of social care, which requires a classify-analysis approach. Using a classify-analysis approach to estimate associations between the latent class variable and other observed variable is straightforward; however, it is known to produce attenuated estimates.^2,3^ To circumvent this limitation, the improved classify-analysis proposed by Bray et al.^2^ was used in this study. The inclusive LCA technique by Bray et al.^2^ has been shown to reduce or even eliminate the bias in estimates when associating the latent variable to the outcome. This approach also has the advantage that it can be performed in all modern statistical software LCA packages through the four proposed steps: 1) determine the optimal number of latent classes by fitting and comparing several models without covariates; 2) re-fit the selected latent class model adding other variables of interest (distal outcome and regression covariates) included as covariates in the LCA to produce posterior probabilities; 3) assign individuals to latent classes, using either the maximum-probability assignment or multiple pseudo-class draws; and 4) treat class membership as observed to perform the desired analysis.

In the first step, a sequence of LCA models was estimated starting with a one-class model and increasing the number of classes up to 10 classes. To ensure that the global, rather than a local maxima was reached, 50 random sets of starting values were used as recommended in the poLCA documentation. To assess the fit of the LC model and determine the optimal number of classes the Bayesian Information Criterion (BIC) and the consistent Akaike Information Criterion (cAIC) were used. These values have shown to be the most reliable when identifying the optimal number of classes. The model with the lowest values for the cAIC and BIC was selected as the best fitting model. In the third step poLCA assigns each participant to one latent class according to the highest computed probability membership using maximum-probability assignment. The latent classes were described according to those variables that characterize them through their estimated class-conditional outcome probabilities, where a variable with a probability above > 0.5 would be considered a characteristics of the referred class. In the fourth step, the assigned latent class is treated as an observed variable to perform the intended analysis.

1. Collins LM, Lanza ST. Latent class and latent transition analysis: With applications in the social, behavioral, and health sciences: John Wiley & Sons; 2009.
2. Bray BC, Lanza ST, Tan X. Eliminating bias in classify-analyze approaches for latent class analysis. Structural equation modeling: a multidisciplinary journal 2015; 22(1): 1-11.
3. Vermunt JK. Latent class modeling with covariates: Two improved three-step approaches. Political analysis 2010; 18(4): 450-69.

**Fit statistics for the LCA model section**

Below the fit statistics, BIC and AIC are displayed for LCA models from one to ten classes. The final model, with the lowest BIC, consists of four classes. This model was compared to that with three classes and the fit of the four-class model was superior over the three-class model.

AIC BIC [,3] [,4]

[1,] 19927.38 20130.55 7.553750e+16 11133.026

[2,] 19359.93 19770.76 3.525905e+15 10473.566

[3,] 19083.63 19702.15 3.578719e+12 10105.273

[4,] 18860.37 19686.56 2.983860e+12 9790.008

[5,] 18780.57 19814.44 1.508343e+12 9618.210

[6,] 18748.50 19990.05 2.030165e+12 9494.140

[7,] 18748.64 20197.87 7.246512e+11 9402.286

[8,] 18719.28 20376.18 7.450815e+12 9280.921

[9,] 18772.19 20636.77 1.350133e+12 9241.835

[10,] 18754.55 20826.80 4.941601e+10 9132.192
