# Supplementary material for: Clustering of non-medical risk factors and the association with duration of social care in pregnant women in highly vulnerable circumstances
Source: Eur J Public Health. 2025 Apr 27;35(3):521–7. doi: 10.1093/eurpub/ckaf062 (PMC12199338; doi:10.1093/eurpub/ckaf062)
Supplement: ckaf062_Supplementary_Data [file ckaf062_supplementary_data.zip › ckaf062_Supplementary_Data/ejph-2024-06-om-0381-File008.docx]

Appendix 3. Primary and sensitivity regression models

| Lineair regression analyses | | |  |  | |  |  | | | | |  |
| --- | --- | --- | --- | --- | --- | --- | --- | --- | --- | --- | --- | --- |
| *Primary regression model * (n=840)* | | |  |  | |  |  | | | | |  |
|  | Crude regression |  | | | Adjusted regression ** | | |  | | |  |  |
|  | *Effect estimate (β), 95%CI* | *p-value* | | | *Effect estimate (β), 95%CI* | | | | | *p-value* |  |  |
| Class 1: Complex | 0.083 (-0.025;0.191) | 0.134 | | | 0.033 (-0.072;0.139) | | | | | 0.536 |  |  |
| Class 2: Educational | 0.120 (0.048;0.192) | ***0.001*** | | | 0.088 (0.020;0.157) | | | | | ***0.012*** |  |  |
| Class 3: Social network | 0.127 (0.034;0.220) | ***0.008*** | | | 0.090 (0.001;0.180) | | | | | ***0.048*** |  |  |
| Class 4: Financial | ***Reference*** |  | | | ***Reference*** | | | | |  |  |  |
| *Sensitivity regression model 1, excluding cases with less than three months of social care* (n=696)* | | | | | | | | | | | | |
|  | Crude regression |  | | | Adjusted regression ** | | | |  | |  |  |
|  | *Effect estimate (β), 95%CI* | *p-value* | | | *Effect estimate (β), 95%CI* | | | | | *p-value* |  |  |
| Class 1: Complex | 0.082 (0.003;0.161) | ***0.041*** | | | 0.145 (-0.058;0.347) | | | | | 0.161 |  |  |
| Class 2: Educational | 0.122 (0.070;0.175) | ***< 0.001*** | | | -0.023 (-0.135;0.089) | | | | | 0.686 |  |  |
| Class 3: Social network | 0.096 (0.030;0.162) | ***0.005*** | | | 0.226 (0.082;0.370) | | | | | ***0.002*** |  |  |
| Class 4: Financial | ***Reference*** |  | | | ***Reference*** | | | | |  |  |  |
| *Sensitivity regression model 2, similar to model 1 but also excluding women that still receive social care* (n=627)* | | | | | | | | | | |  |  |
|  | Crude regression |  | | | Adjusted regression ** | | | | |  |  |  |
|  | *Effect estimate (β), 95%CI* | *p-value* | | | *Effect estimate (β), 95%CI* | | | | | *p-value* |  |  |
| Class 1: Complex | 0.081 (-0.002;0.165) | ***0.057*** | | | 0.045 (-0.034;0.124) | | | | | 0.267 |  |  |
| Class 2: Educational | 0.118 (0.063;0.174) | ***< 0.001*** | | | 0.090 (0.037;0.142) | | | | | ***< 0.001*** |  |  |
| Class 3: Social network | 0.094 (0.024;0.165) | ***0.009*** | | | 0.074 (0.007;0.141) | | | | | ***0.030*** |  |  |
| Class 4: Financial | ***Reference*** |  | | | ***Reference*** | | | | |  |  |  |
| ** Class 4 used as a reference ** Adjusted for maternal age, deprived neighbourhood and social care provider* | | | | | | | | | | | | |
